# Audit of outcomes of early initiation of antiretroviral therapy in children admitted to the paediatric wards at Kilifi district hospital, Kenya

**DOI:** 10.1186/1758-2652-13-S4-P156

**Published:** 2010-11-08

**Authors:** HM Nabwera, S Mwaringa, R Kimutai, JA Berkley

**Affiliations:** 1 KEMRI-Wellcome trust research programme, P.O.BOX 230, Kilfi, Kenya; 2 KEMRI-Wellcome trust research programme, Kilifi, Kenya

## Background

Studies in adults suggest that early initiation of anti-retroviral therapy (ART), during treatment of those presenting with opportunistic infections, results in better outcomes than deferred treatment. However, data on the effects of early treatment of children are sparse. Kilifi district hospital (KDH) serves a population of over 250,000. It provides HIV services and ART. All children admitted to the paediatric wards at KDH are routinely tested for HIV.

## Purpose of the study

To ascertain the outcomes of early initiation of ART in children in a rural African setting around the time of an acute admission.

## Methods

We retrospectively reviewed inpatient and clinic records of all HIV antibody positive children admitted to the paediatric wards at KDH between May 2007- December 2008. The demographic surveillance system was used to ascertain outcomes.

## Summary of results

Over the 19-month period, there were 9,377 admissions to the paediatric wards. Of these 407 (5.6%) children had a positive HIV antibody test. 47 children met criteria for starting ART according to Kenyan national guidelines. 22 (47%) of these children started ART in the acute phase, and a further 13 (28%) started subsequently in clinic, but 12 (25%) were lost to follow-up before ART initiation. The median age was 2.9 years (IQR 1-3.9 years). The median CD4 percentage was 11% (IQR 7.6%-15.5%). There was a high rate of default from follow-up even after initiation of ART (40%). Most of the children who remained in care were still alive 19 (90.5%) and had attained nutritional recovery (87%) with a minority having adverse events (13%) 6 months after initiation of ART. Comparing acute versus deferred initiation groups, those started early were younger and had lower baseline CD4 percentages. There was no difference between the 2 groups in terms of incidence of adverse events or survival to 6 months after starting treatment.

## Conclusions

Our data suggests that commencing ART in children around the time of an acute admission even in children who are severely malnourished may be safe and result in nutritional recovery with good survival. However, due to the high number of defaulters, we were unable to draw definitive conclusions comparing early and deferred initiation. We recommend that defaulter tracing should be an integral part of all ART programmes, and randomised studies to compare timing of initiation in larger cohorts.

